# 5‐Hydroxymethylcytosine Profiles of cfDNA in Urine as Diagnostic, Differential Diagnosis and Prognostic Markers for Multiple Myeloma

**DOI:** 10.1002/cam4.70477

**Published:** 2024-12-23

**Authors:** Weiwei Xie, Xuehui Li, Hangyu Chen, Jinlin Chu, Lei Zhang, Bo Tang, Wenrong Huang, Linlin Li, Jian Lin, Yujun Dong

**Affiliations:** ^1^ Department of Hematology Peking University First Hospital Beijing People's Republic of China; ^2^ Department of Pharmacology Xinjiang Medical University Urumqi China; ^3^ Department of Pharmacy Peking University Third Hospital Beijing China; ^4^ Synthetic and Functional Biomolecules Center Peking University Beijing China; ^5^ Key Laboratory of Tropical Biological Resources of Ministry of Education, School of Pharmaceutical Sciences Hainan University Haikou China; ^6^ Peking University Third Hospital Cancer Center Beijing China; ^7^ Department of Hematology Fifth Medical Center, General Hospital of the People's Liberation Army Beijing People's Republic of China; ^8^ Key Laboratory of Active Components of Xinjiang Natural Medicine and Drug Release Technology Urumqi China

**Keywords:** 5‐hydroxymethylcytosine (5hmC), diagnostic, differential diagnosis, multiple myeloma, prognostic, urine

## Abstract

**Background:**

An effective urine‐based method for the diagnosis, differential diagnosis and prognosis of multiple myeloma (MM) has not yet been developed. Urine cell‐free DNA (cfDNA) carrying cancer‐specific genetic and epigenetic aberrations may enable a noninvasive “liquid biopsy” for diagnosis and monitoring of cancer.

**Methods:**

We first identified MM‐specific hydroxymethylcytosine signatures by comparing 64 MM patients, 23 amyloidosis (AM) patients and 59 healthy cohort. Then, we applied a machine learning algorithm to develop diagnostic and differential diagnosis model. Finally, the prognosis of MM patients was predicted based on their survival time at the last follow‐up.

**Results:**

We identified 11 5hmC markers using logistic regression algorithm could effectively diagnosis MM (AUC = 0.902), and achieved 85.00% specificity and 85.71% sensitivity. These 11 markers could also effectively differential diagnosis MM (AUC = 0.805) with 88.89% specificity and 73.08% sensitivity. In addition, the prognostic prediction model also effectively predicted the prognosis of patients with MM (*p* < 0.01), of which 4 differential markers (*RAPGEF2*, *BRD1*, *TET2*, *TRAF3IP2*) could independently predict the prognosis of MM.

**Conclusions:**

Together, our findings showed the value of urine cfDNA hydroxymethylcytosine markers in the diagnosis, differential diagnosis and prognosis of MM. Meantime, our study provides a promising and completely non‐invasive method for the diagnosis, differential diagnosis and prognosis prediction of MM.

**Abbreviations**
5hmC: 5‐hydroxymethylcytosine
5mC: 5‐methylcytosine
AUC: the area under ROC curves
cfDNA: cell‐free DNA
DhMR: 5hmC differentially modified region
HR: hazard ratio
MM: multiple myolema
QC: quality control
ROC: receiver operating characteristic
TET: ten‐eleven translocation
TSS: transcription start site
TTS: transcription termination site
wd‐score: weighted diagnostic score

## 1 Introduction

Multiple myeloma (MM), a plasma‐cell malignancy, is the second common malignant tumor of the blood system, characterized by expansion of malignant plasma cells (PCs) in the bone marrow (BM), leading to destruction of the function of major organs [1]. At present, the main diagnostic methods for MM are BM aspiration to detect the proportion of PCs or tissue biopsy to prove plasma cell tumor [2, 3]. However, early symptoms of MM are not obvious and the clinical manifestations in later stages are diverse and complex, interference from asymptomatic MM patients and other malignant plasma cell diseases can result in misdiagnosis or delayed diagnosis [4], further affecting the prognosis of the disease. In addition, traditional puncture biopsy methods have significant trauma, and existing liquid biopsy methods lack precise diagnostic biomarkers [5]. Therefore, a clinically convenient, non‐invasive liquid biopsy method and the search for more accurate biomarkers are crucial for the diagnosis and differential diagnosis of MM. Meantime, despite advances in the treatment of MM, primary treatment resistance, as well as early relapse after upfront therapy, are strongly associated with poor outcomes and reduced survival [6]. Clinically, a new method and new biomarkers are necessary to predict the prognosis of patients.

In recent years, non‐invasive detection of cell‐free DNA (cfDNA) in the circulation system has been used for early detection of various cancers [7]. In the current study, cfDNA is thought to derive primarily from the apoptosis of normal cells of the hematopoietic system, such as white blood cells (55%), red blood cell progenitors (30%), vascular endothelial cells (10%), and liver cells (1%) [8]. When cells die, cfDNA is released into the circulatory system, and some of it passes through the filtration barrier of the glomeruli into the urine, which can comprehensively reflect the state of an individual [9, 10, 11]. Compared with other liquid biopsy methods, urine cfDNA has the advantages of being completely non‐invasive and facilitating repeated sampling, and the proportion of cfDNA in urine is higher than in plasma [12]. Havell Markus et al. [11] found that certain regions of the genome in healthy people's urine are protected, but the same regions in cancer patients have more fragmentation and may be used for cancer diagnosis. Pradeep S. Chauhan et al. [13] found that urine cfDNA can sensitively detect molecular residual disease in bladder cancer patients and accurately predict survival, and is far superior to plasma circulating tumor DNA (ctDNA). 5‐hydroxymethylcytosine (5hmC) is a stable epigenetic marker, which is a relatively stable product in the process of 5‐methylcytosine (5mC) demethylation [14] and can be used as a biomarker for diagnosing various human cancers [15, 16, 17, 18]. Brian C.‐H. Chiu et al. [19] found that detection of plasma cfDNA 5hmC epigenetic modifications can be better to differential diagnosis of MM, and can embody the MM race specific ways in the process of development. However, there is currently limited research on using 5hmC biomarkers in urine cfDNA for the diagnosis of MM, which may serve as a more valuable non‐invasive diagnostic method.

In this study, we obtained the whole‐genome 5hmC profiles of urine cfDNA from 59 healthy cohort, 64 MM patients and 23 amyloidosis (AM) patients using the 5hmC‐Seal technology. Compared the distribution differences of 5hmC signals among different genomes, analyzed the differential 5hmC biomarkers associated with MM, and selected 5hmC biomarkers to construct diagnostic and differential diagnosis model for MM using machine learning algorithms, and the prognosis of MM patients was predicted based on their survival time at the last follow‐up visit. This study provides a novel method for diagnostic, differential diagnosis and prognostic of MM patients.

## 2 Materials and Methods

### 2.1 Study Participants

All samples were collected at Peking University First Hospital and the Chinese People's Liberation Army General Hospital. MM and AM samples were collected from newly diagnosed MM and AM patients and cryopreserved together with healthy control samples at −80°C until use. Written informed consent was obtained from all subjects in accordance with the Declaration of Helsinki before the study, and the study has been approved by the Institutional Review Board of PKUFH (No. 2022 yan 196‐002). The inclusion criteria were based on the diagnostic criteria issued by the International Myeloma Working Group (IMWG) patient history [20], procedural details, and other relevant clinical and diagnostic information were collected using case report forms. All patients were followed from the date of diagnosis until death or the last follow‐up date.

### 2.2 Samples Collection and cfDNA Preparation

Urine specimens were collected into 50 mL centrifuge tubes (DNase/RNase). Centrifuge tubes were maintained at 15°C–25°C with urine separation performed within 24 h by centrifugation of urine at 3000 ×*g* for 15 min at 4°C, followed by transfer of the urine supernate to a new tube. Add 70 μL urine conditioning buffer for every 1 mL of urine, mix the urine mixture well by vortexing (after adding and mixing urine with urine conditioning buffer, urine can be stored up to 1 month at ambient temperature). Urine was aliquoted for subsequent cfDNA isolation or storage at −80°C. The urine cfDNA was extracted using the Quick‐DNA Urine Kit (ZYMO) following the manufacturer's protocol and then stored at −20°C.

### 2.3 5hmC‐Seal Library Construction and Sequencing

The 5hmC library of urine samples was constructed according to the previously reported method [21]. The high‐efficiency hmC‐seal technique was used with 10 ng of urine cfDNA loading. Extracted cfDNA was end‐repaired with the KAPA Hyper Prep Kit (KAPA Biosystems) and ligated to adapters compatible with Illumina sequencing. The biotinylation of 5hmC was performed in two processes, first the glycosylation reaction was carried out at 37°C for 2 h, in order to link the UDP‐6‐N3‐Glc molecule on the 5‐hydroxyl bond, and purified by the DNA Clean & Concentrator Kit (ZYMO).

Next, the purified DNA was then incubated with DBCO‐PEG4‐Biotin for 2 h at 37°C and ligated for a second purification. The biotinylated DNA fragments were then enriched by binding streptavidin beads (Life Technologies). The enriched DNA fragments were amplified by PCR, and finally the PCR products were purified with the use of AMPure XP beads (Beckman). Library concentration was determined using a Qubit 4.0 fluorometer (Life Technologies). Sequencing was performed at 5hmC on a NextSeq 500 platform based on paired 39‐bp high‐throughput sequencing.

### 2.4 Mapping and Identifying 5hmC‐Enriched Regions

The constructed libraries were evaluated for sequence quality using FastQC (version 0.11.5). The raw 5hmC data obtained from sequencing were aligned to the human genome (version hg 19) with bowtie2 (version 2.2.9) [22], and further filtered with SAMtools (version 1.3.1) [23], to retain unique non‐duplicate matches to the genome. Pair‐end reads were extended and converted into BedGraph format using Bedtools (version 2.19.1) [24], and then converted to bigwig format for visualization using bedGraphToBig‐Wig from Integrated Genomics Viewer. MACS2 (version 2.1.1) was used to identify potential 5hmc‐enriched regions in each sample [23]. Regions that were present in more than 10 samples and less than 1000 bp in length were combined into one uniform catalog for each patient. Genomic regions that tended to show artifact signals according to ENCODE were filtered out. The 5hmc‐enriched regions were generated by intersecting individual peak calling files with the merged peak files. We used the CHIP seeker package to annotate the 5hmc‐enriched region, and the genes closest to this region were used for the following analysis.

### 2.5 Functional Analysis Approach

Differences in gene function analysis through the GO/KEGG annotation platform website (http://123.57.212.209:8899/) and Metascape (https://metascape.org/) [25].

### 2.6 Construction of Diagnostic Model and Differential Diagnosis Model

MM patients were randomly divided into training group and validation group according to the ratio of 2:1. The train_test_split of Scikit‐Learn (version 0.22.1) [26] package in Python (version 3.6.10) was used to establish the diagnostic model. In the training group, the 5hmC differentially modified regions (DhMRs) was identified by the EdgeR package (version 3.24.3) [27] in Rstudio (version 3.5.0) according to the filtering threshold (*p* < 0.01 and |log 2FoldChange| > 0.5). Then, the DhMRs were further filtered using the recursive feature elimination algorithm (RFECV) in Scikit‐Learn (parameters used: estimator = LogisticRegressionCV (class_weight = ‘balanced’, cv = 2, maxiter = 1000), scoring = ‘accuracy’). Then we trained the logistic regression CV model (LR) with the feature markers obtained in the previous step (parameters used: Maxiter = 100, method = “lbfgs”). The model was used to predict the diagnostic performance of the validation group. In addition, 23 patients with AM were included to verify the effect of the model in the differential diagnosis of MM and AM patients. Receiver operating characteristic (ROC) analysis was used to evaluate the diagnostic performance of the model. The area under the curve (AUC), optimal cutoff, sensitivity, and specificity values were calculated using the sklearn metrics module.

### 2.7 Prognostic Prediction Analysis

Samples of MM patients were grouped according to the median survival. By Rstudio (3.5.0 version) of the DESeq2 package (version 1.30.0) for the analysis of difference in two groups of patients with gene (filter condition: *p* < 0.05 and |log 2 foldchange| > 0.5). Then, the recursive feature elimination algorithm (RFECV) in Scikit‐Learn was used (Parameter: Estimator = LogisticRegressionCV (class_weight = “balance”, cv = 2, maxiter = 1000), score = “accuracy”) to screen differential genes, Screening to gene through Sangerbox website (http://www.sangerbox.com/tool) [28] Hazard thewire (HR), in the end, through Sangerbox website on HR value statistically significant genes individually survival curve is drawn.

### 2.8 Wd‐Score and Wp‐Score

A weighted diagnostic score (wd‐score) was calculated as the sum of the gene‐wise product of logistic model coefficients and corresponding 5hmC marker value for each individual:

$$\mathrm{wd}-\text{score}=\sum_{k=1}^{n}\beta_{k}*{\text{gene}}_{k}$$

where *β*_k_ is the coefficient from the final multivariable logistic model for the kth marker gene, and gene_k_ is the 5hmC level of the kth marker gene.

A weighted prognostic score (wp‐score) was calculated in the same way as the wd‐score, using the formula:

$$\mathrm{wp}-\text{score}=\sum_{k=1}^{n}\beta_{k}*{\text{gene}}_{k}$$

where *β*_k_ is the coefficient from the multivariate logistic model for gene_k_, and gene_k_ is the normalized count of kth marker gene in the final panel.

### 2.9 Statistical Analysis

For basic information and clinical data, they are represented by the mean ± standard deviation and the median. The difference analysis of clinical indicators between the two groups is conducted through *t* test and Wilcoxon test. Subsequent data analysis is performed using R version 4.1.0. The glm function in R‐base and the pROC package in RStudio version 1.15.3 are used to analyze the ability of clinical data to predict survival.

## 3 Results

### 3.1 Clinical Characteristics of Patients

The basic information and clinical indicators of 59 healthy, 64 MM patients and 23 AM patients are shown in Table 1. There were no significant differences in age, sex, and overall survival between the MM and AM groups. In addition, MM patients were divided into stages I, II and III according to Durie–Salmon (DS) stages. The basic information and clinical indicators of patients with different stages were shown in Table 2. As the disease progressed, the levels of bone marrow plasma cell, serum M protein region, and serum free light chains‐kappa were gradually upregulated.

**TABLE 1:** Characteristics of the study subject.

Characteristics	MM (*n* = 64)	AM (*n* = 23)	Healthy (*n* = 59)
Age (mean (SD))	62.0 (10.3)	58.1 (7.7)^a^	31.2 (1.4)^****^
Gender (male/female)	40/24	15/8^a^	11/48*
Overall survival (mean (SD) month)	18.60 (7.7)	19.0 (2.8)^a^	—
Serum calcium (median mmol/L)	2.37	NA	—
Serum creatinine (median μmol/L)	94.77	88.65***	—
Glomeruar filtration rate (median mL/min)	59.92	76.92^a^	—
Hemoglobin (median g/L)	98.50	NA	—
Total protein (median g/L)	71.60	55.75***	—
Serum albumin (median g/L)	37.10	24.90^****^	—
24 h‐urinary albumin excretion (median g/d)	1.80	6.30^****^	—
Bone marrow plasma cell (median %)	11.00	11.00*	—
Serum M protein region (median %)	8.30	9.29^a^	—
Serum free light chains‐kappa (median mg/L)	16.70	21.00^a^	—
Serum free light chains‐lambda (median mg/L)	30.60	64.30^a^	—

*Note:* Data are presented as mean ± SD (standard deviation) or median.

Abbreviations: AM, amyloidosis; MM, multiple myeloma.

^a^ No significance.

* *p* < 0.05.

*** *p* < 0.001.

**** *p* < 0.0001.

**TABLE 2:** Durie–Salmon staging of MM patients.

MM (*n* = 64)	I (*n* = 8)	II (*n* = 7)	III (*n* = 49)
Age (mean (SD) year)	60.25 (4.3)	63.00 (3.0)	62.14 (1.5)
Gender (male/female)	7/1	5/2	28/21
Overall survival (mean (SD) month)	20.13 (3.9)	17.71 (1.5)	18.46 (1.1)
Bone marrow plasma cell (median %)	10.00	10.50	14.00
Serum M protein region (median %)	0.07	3.95	16.70
Serum free light chains‐kappa (median mg/L)	9.79	10.30	16.70

*Note:* Data are presented as mean ± SD (standard deviation) or median.

Abbreviation: MM, multiple myeloma.

### 3.2 Genomic Distributions of 5hmC in Multiple Myeloma

**FIGURE 1:**
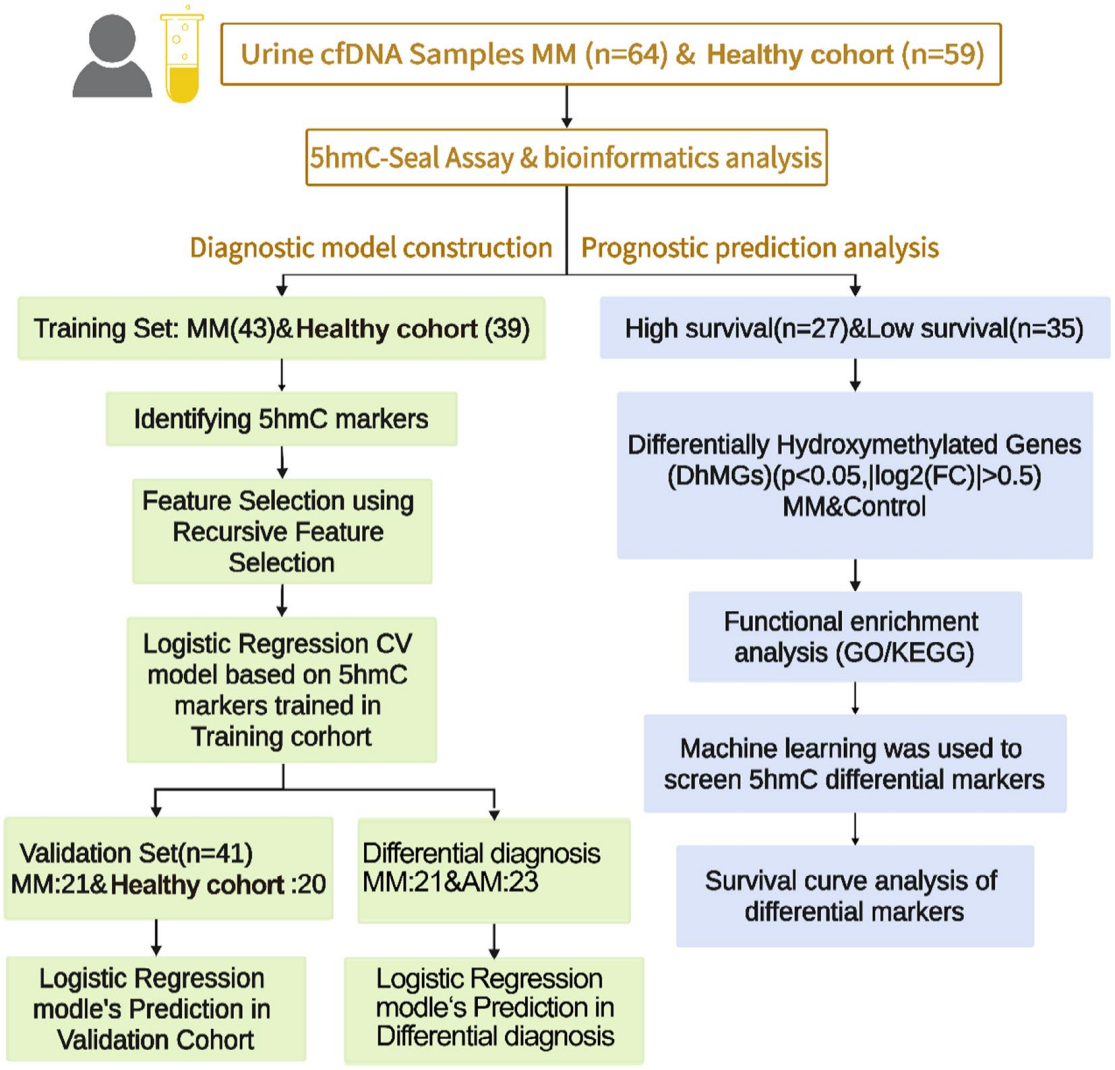
Overview of study design. Candidate differential markers between healthy cohort and MM patients were identified by 5hmC‐Seal. Left panel: diagnostic marker selection: LogisticRegressionCV analyses were applied to a training cohort of 43 MM patients and 39 healthy cohort to identify a final selection of markers. These markers were used to validate a validation cohort of 21 MM patients and 20 healthy cohort, and a differential diagnosis cohort of 23 AM patients and 21 MM patients. Right panel: prognostic marker selection: UniCox and LASSO Cox were applied to 62 MM patients with survival data to identify a final selection of markers.

**FIGURE 2:**
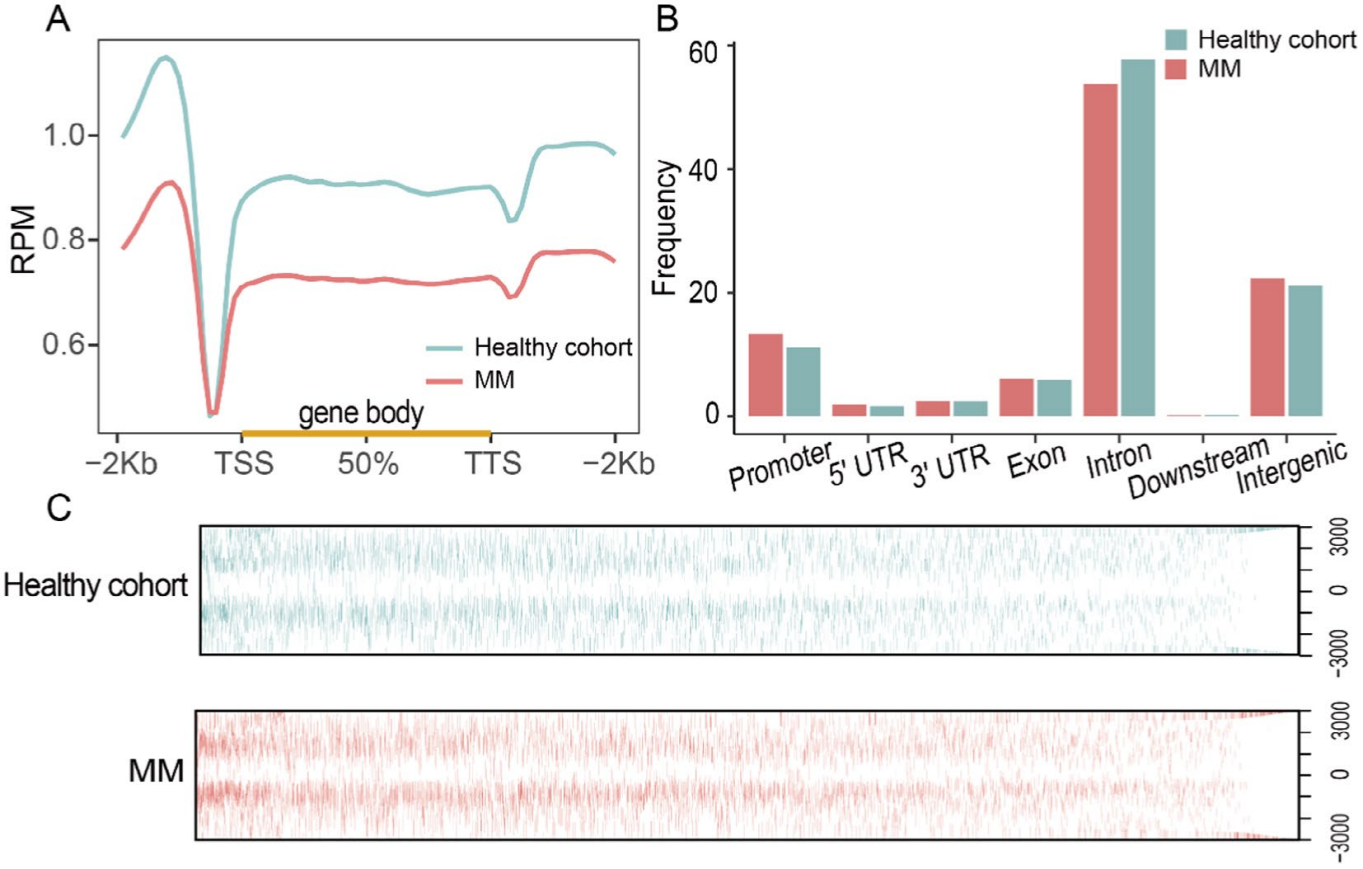
Characteristics of 5hmC distribution in plasma cfDNA of healthy cohort and MM patients. (A) The profiled 5hmC‐Seal data in all samples cfDNA are enriched in gene bodies and depleted in the flanking regions. (B) Genome‐wide 5hmC distribution in different genomic features (healthy cohort vs. MM). (C) 5hmC modification between −3 kb and 3 kb of the TSS increased with MM patients and healthy cohort.

cfDNA was extracted from 59 healthy cohort and 64 MM patients urine samples for library construction, quality control (QC), and 5hmC sequencing, map the genome‐wide 5hmC profiles for all samples. According to an analysis of quality control of urine cfDNA fragments, healthy cohort and MM patients cfDNA fragment size all around 150 bp (Figures 1B and S1A), consistent with existing research [29]. The results of sequencing showed that a total of 43,980 sites were annotated, 5hmC signal intensity of the control group was higher than that of the MM patients (Figure 2A), and the 5hmC signals were mainly distributed in the transcription start site (TSS), transcription termination site (TTS), intron, intergenic and promoter of the genome (Figure 2B). Meantime, although the total number of peaks decreased in MM patients, the 5hmC modification between −3 kb and 3 kb of the TSS increased with MM patients (Figure 2C). This is consistent with previous report [15], suggesting that the level of 5hmC signaling is related to the transcriptional activity of genes. The results of venn diagram showed that the 5hmC sites in MM patients and healthy cohort were mostly different in genebody, which laid a foundation for us to find 5hmC markers for the diagnosis of MM patients (Figure S2).

### 3.3 Identified DhMRs in Multiple Myeloma and Function Exploration

**FIGURE 3:**
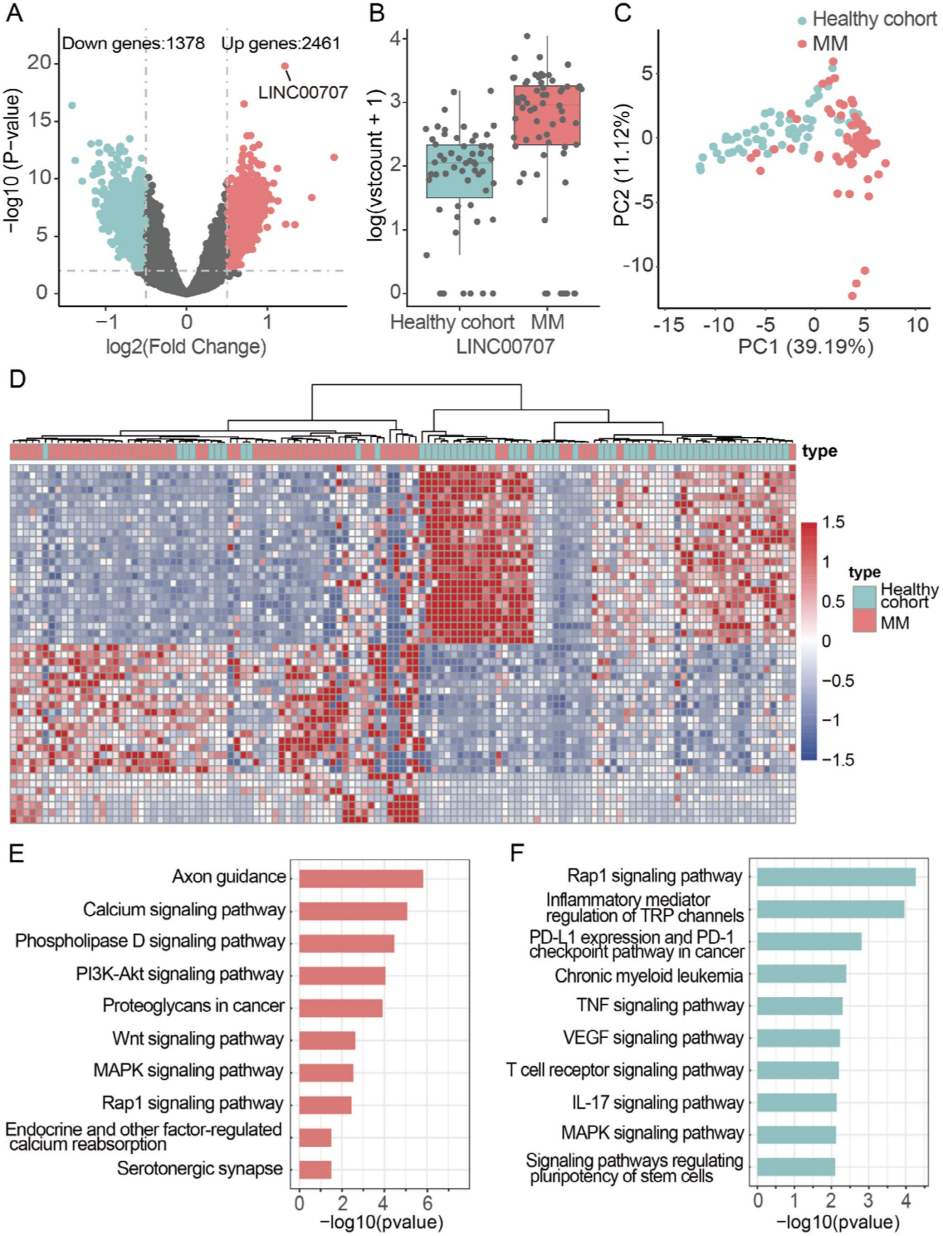
Analysis of 5hmC differences in urina cfDNA between healthy cohort and MM patients. (A) Volcano map. The discrepancies loci in the healthy cohort and MM patients (|log2FC| > 0.5, *p* < 0.05) have been highlighted in red (up) or green (down), gray dot represents no differentially expressed loci. (B) Boxplot of LINC00707 (healthy cohort vs. MM). (C) PCA differentiation between healthy cohort and MM patients (healthy cohort in green, MM patients in red). (D) Heatmap of top 50 DhMGs with sample type and age information labeled. Unsupervised hierarchical clustering was performed across genes and samples. (E) Functional analysis of sites with high hydroxymethylation levels. (F) Functional analysis of sites with low hydroxymethylation levels.

First, we conducted differential analysis between healthy cohort and MM patients, with 3839 DhMRs (Table S1) in MM patients compared to the healthy cohort, including 2461 sites with high levels of DhMGs and 1378 sites with low levels of DhMGs (Figure 3A). The hydroxymethylated regions (hMRs) with the most significant *p*‐value was annotated to gene LINC00707, which showed significantly higher hydroxymethylation levels in MM patients than healthy cohort (Figure 3B). The results of principal component analysis (PCA) showed that the healthy cohort and MM patients could be separated (Figure 3C), and the heatmap also showed that the two groups could be distinguished by unsupervised cluster analysis of the top 50 differential markers (Figure 3D). Functional annotation of differential markers of high and low levels of DhMGs were performed separately. The results showed that they were all enriched in some disease‐related pathways (Figure 3E,F). GO analysis showed that all differential 5hmC markers were enriched in Wnt, GTPase, kidney development and other functionally related pathways (Figure S3A), and KEGG functional analysis showed that PI3K‐Akt, Ras, MAPK, and T cell were enriched receptor and other disease‐related pathways (Figure S3B). CNET network analysis showed marked difference between two groups of 5hmC enrichment in Axon guidance, calcium signaling and ECM receptor pathway (Figure S3C).

### 3.4 Establishing a Diagnostic Model of Multiple Myeloma Use DhMRs

**FIGURE 4:**
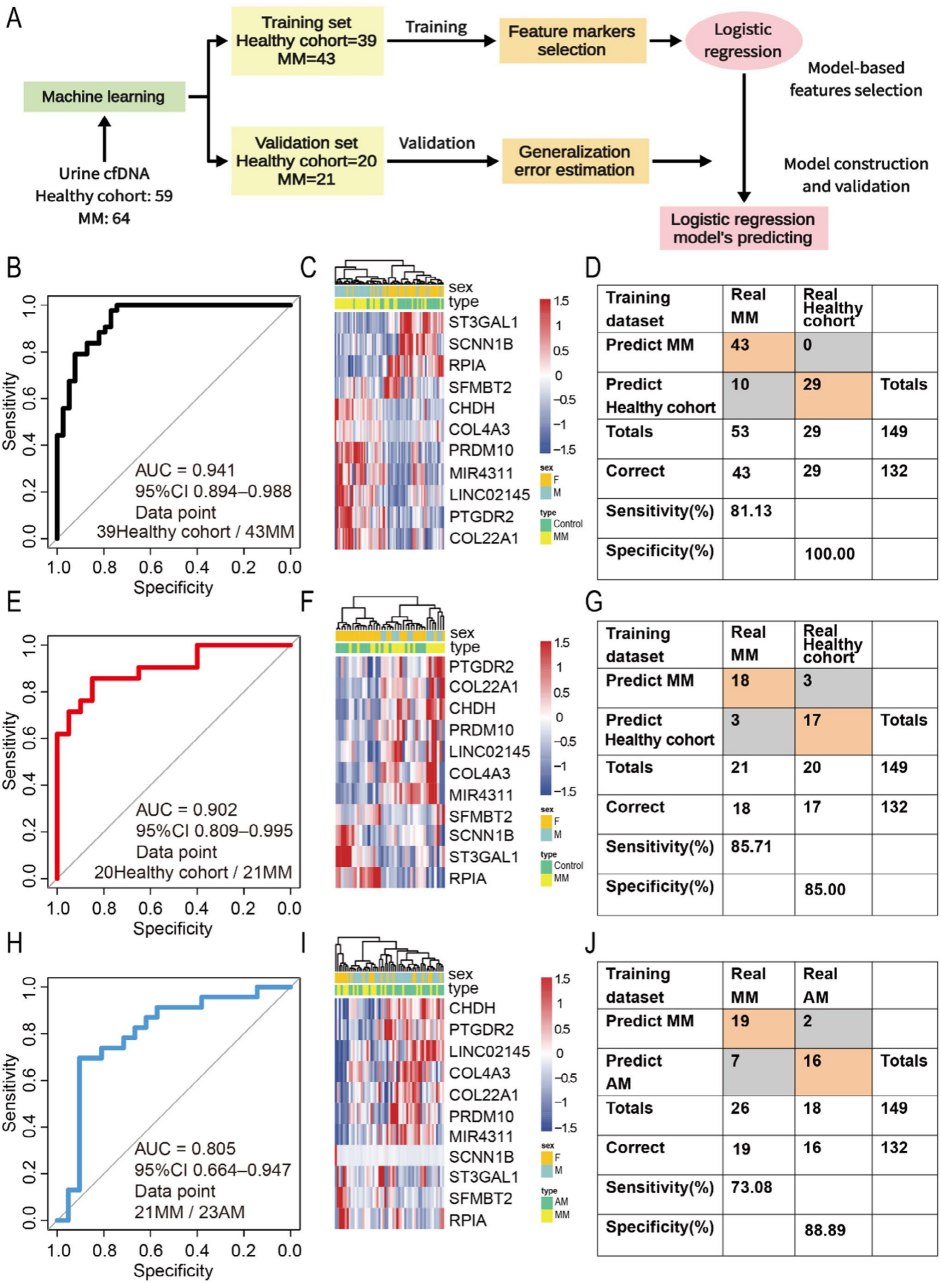
Diagnostic accuracy of urine cfDNA 5hmC marker in training and validation cohorts. (A) Overview of diagnostic model design. (B) ROC analysis of the diagnostic accuracy of 11 markers obtained by machine learning in the training group. (C) Heatmap of the 11 markers in the training group. (D) Specificity and sensitivity of the diagnosis of 11 markers in the training group. (E) ROC analysis of the diagnostic accuracy of 11 markers in the validation group. (F) Heatmap of the 11 markers in the validation group. (G) Specificity and sensitivity of the diagnosis of 11 markers in the validation group. (H) ROC analysis of the diagnostic accuracy of 11 markers in the differential diagnosis group. (I) Heatmap of the 11 markers in the differential diagnosis group. (J) Specificity and sensitivity of the diagnosis of 11 markers in the differential diagnosis group.

To further evaluate the potential of the differential 5hmC profile of urine cfDNA as diagnostic markers for MM, we divided the samples into training and validation cohorts at a ratio of 2:1 to construct the diagnostic model (Figure 4A). By using the recursive feature elimination algorithm based on logistic regression CV estimator, 11 differential markers were obtained, which could effectively diagnose the training (AUC = 0.941) and validation cohorts (AUC = 0.902) (Figure 4B,E), achieving 100.00% specificity and 81.13% sensitivity in the training cohort (Figure 4D) and 85.00% specificity and 85.71% sensitivity in the validation cohort (Figure 4G). To test the differential diagnosis of these 11 5hmC markers for MM and other similar diseases, we included an additional 23 patients with AM. The analysis results showed that the AUC value of 11 markers was 0.805 (Figure 4H), achieving 88.89% specificity and 73.08% sensitivity (Figure 4J). Unsupervised clustering showed that these 11 markers could distinguish MM patients from healthy cohort in the training and validation cohorts (Figure 4C,F), and can also distinguish MM patients from AM patients (Figure 4I). Finally, we also calculated the individual AUC for each of the 11 5hmC markers in the training (Figure S6), validation (Figure S7) and differential diagnosis cohorts (Figure S8).

Next, we analyzed the score of 11 5hmC difference markers in each sample after machine learning screening, analysis showed that the wd‐score of MM patients was significantly higher than that of the healthy cohort (Figure S4A). MM is an elderly disease, and it can be seen from the results that the older the patients are, the higher the wd‐score is (Figure S4B). The 64 patients with MM included in this study were in different disease stages, but the wd‐scores of patients with different stages were higher than those of the control group (Figure S4C). Some MM patients would induce renal injury in the later stage of the disease. There was no significant difference in wd‐score between patients with and without renal injury, but the wd‐score was higher than that of the healthy cohort (Figure S4D). In addition, the wd‐score of men was also higher than that of women (Figure S4E). The score of these 11 5hmC markers in each sample was able to differentiate MM patients from the healthy cohort with an AUC value of 0.923 (Figure S4F). In addition, the weighted differential diagnosis scores (wdd‐score) of healthy cohort, MM patients and AM patients in the differential diagnosis cohort were compared, and the scores of AM patients were significantly higher than those of MM patients (Figure S4G). The score of these 11 5hmC markers in each sample was able to differentiate MM patients and AM patients with an AUC value of 0.810 (Figure S4H).

Additionally, to further validate the accuracy of the diagnostic model, we conducted a validation using the dataset GSE146649 from the NCBI Gene Expression Omnibus (GEO) database. The diagnostic accuracy was 0.742 (Figure S5A), with a sensitivity and specificity of 58.33% and 90.00%, respectively (Figure S5B). Moreover, the wd‐score trends between the healthy cohort and the MM group were consistent with the aforementioned results (Figure S5C). These results indicate that urine cfDNA‐based 5hmC markers are a promising diagnostic and differential diagnosis tool for MM.

### 3.5 Prognostic Prediction of Multiple Myeloma Based DhMRs

**FIGURE 5:**
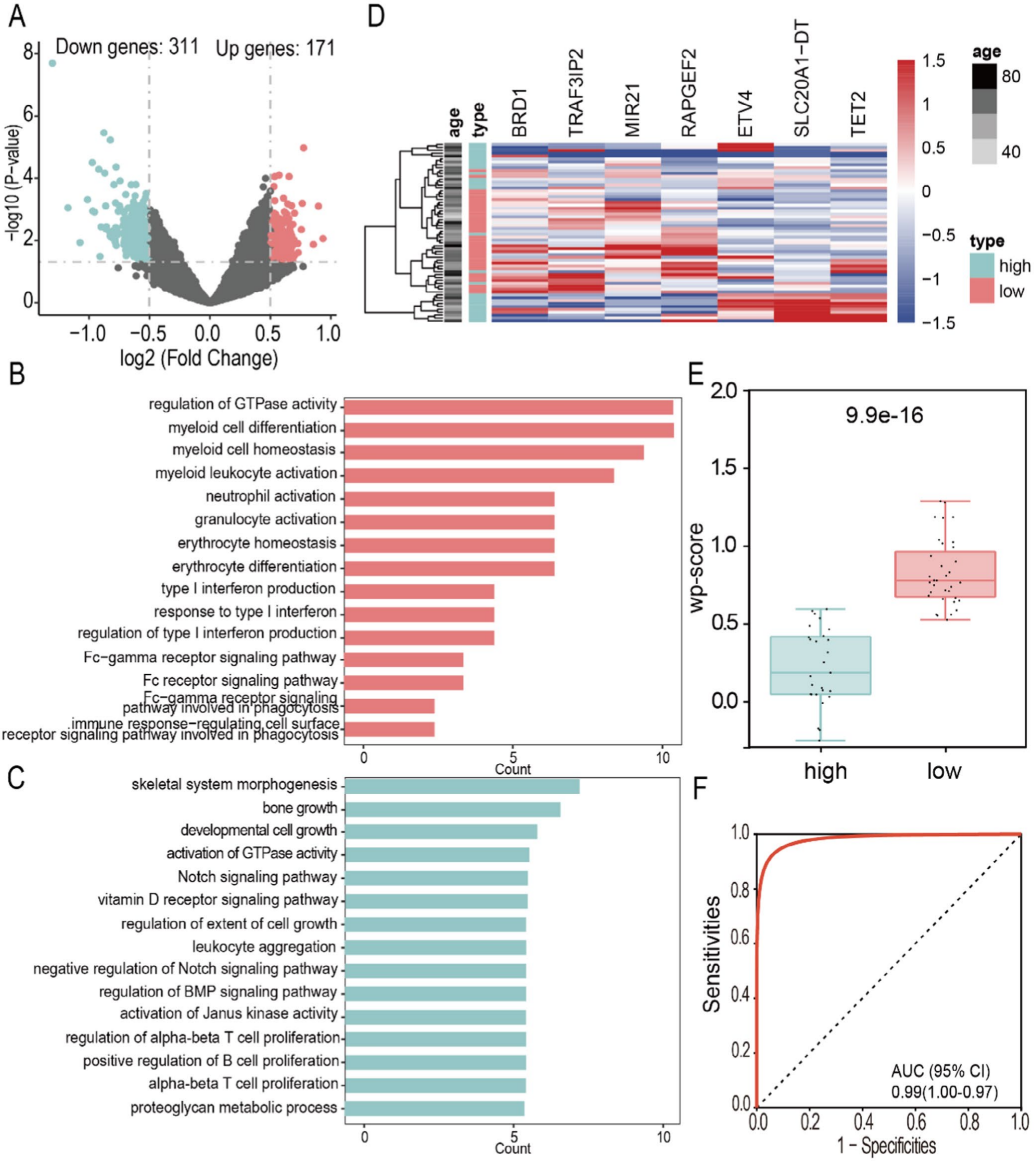
The difference between long survival and short survival of MM patients. (A) Volcano map. The discrepancies loci in the high and low survival time patients (|log2FC| > 0.5, *p* < 0.05) have been highlighted in red (up) or green (down), gray dot represents no differentially expressed loci. (B) Functional analysis of sites with high hydroxymethylation levels. (C) Functional analysis of sites with low hydroxymethylation levels. (D) Heatmap of the 7 markers in patients with high survival and low survival. (E) Comparison of wp‐score between high and low survival time patients. (F) ROC analysis was used to show the accuracy of the weighted diagnostic scores of the 7 differential markers in distinguishing patients with high survival and low survival.

**FIGURE 6:**
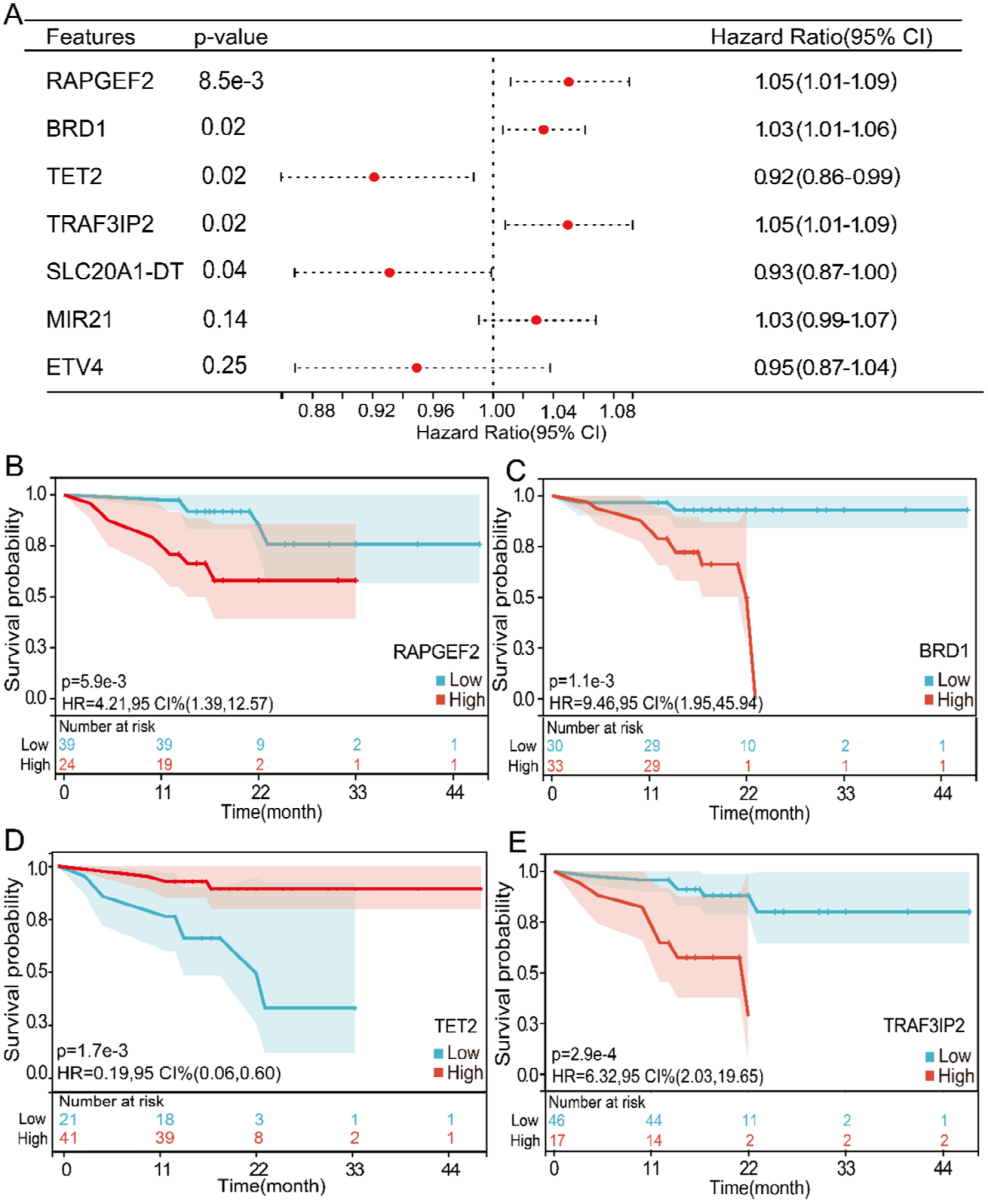
Prognostic prediction of MM survival based on 5hmC markers. (A) HR forest plot of 7 5hmC markers obtained by machine learning through multivariate Cox regression analysis. (B–E) Kaplan–Meier survival curves for *RAPGEF2*, *BRD1*, *TET2* and *TRAF3IP2* between high and low survival patients.

Taking the survival period at the last follow‐up of each patient as the event endpoint, according to the median overall survival (OS) (18 months), all MM patients were divided into two groups: short OS (OS < 18 months, *n* = 35) and long OS (OS ≥ 17 months, *n* = 27), two samples was excluded because its OS was unknown. First, we observed 171 high DhMRs and 311 low DhMRs in short OS patients when compared with those in long OS (Figure 5A, Table S2). Functional annotation of DhMGs showed that high hydroxymethylation genes were enriched in GTPase activity, myeloid cells, myeloid leukocytes, FC receptor and other disease‐related pathways (Figure 5B), while low hydroxymethylation genes were mainly enriched in skeletal system development, T cells, B cells and other pathways (Figure 5C). This suggests that the growth and development of the skeletal system as well as inflammatory and immune responses may be the main factors affecting the quality of life in MM. The UniCox and LASSO Cox methods were implemented to reduce the dimensionality, and a Cox‐model was constructed to predict prognosis with a seven‐marker panel. We found that these 7 markers could distinguish long OS patients and short OS patients (Figure 5D). The weighted prognosis score of patients with low survival was significantly higher than that of patients with high survival (Figure 5E), and the two groups of patients could be distinguished with an accuracy of 0.99 AUC (Figure 5F). The hazard ratio (HR) analysis of routine clinical tests showed that the HR values of serum albumin and serum calcium were statistically significant. Serum albumin level was positively correlated with the survival time of MM patients, while serum calcium level was negatively correlated with the survival time of MM patients (Figure S9A). ROC analysis showed that the accuracy of each clinical marker alone in predicting the survival of MM patients was significantly lower than that of 5hmC differential markers (Figure S9B). The HR values of the 7 markers obtained by screening were also analyzed and the *p* values of *RAPGEF2*, *BRD1*, *TET2*, *TRAF3IP2* and *SLC20A1‐DT* were statistically significant (Figure 6A). The first four markers with statistically significant differences in *p* values were analyzed separately by Kaplan–Meier survival curves for *RAPGEF2*, *BRD1*, *TET2* and *TRAF3IP2*, the results showed that with the increase of 5hmC levels of *RAPGEF2*, *BRD1*, and *TRAF3IP2*, the OS of patients with MM was shorter, the increase of 5hmC levels of *TET2*, the OS of patients with MM was longer (Figure 6B–E).

## 4 Discussion

Although previous studies have reported that the 5hmC modification of plasma cfDNA can be used as a potential prognostic marker in MM patients [30], the use of urine cfDNA in MM diagnosis, differential diagnosis and prognosis prediction has not been fully studied. Liquid biopsy technology has been proved to be a promising method. Compared with the current common BM puncture and blood drawing tests, urine biopsy as a large, convenient sampling method can be repeated for a long time, and can achieve completely non‐invasive sampling. Current studies have shown that cfDNA is mainly derived from the apoptosis of hematopoietic cells such as white blood cells and red blood cell progenitors. These cells are prolifically born in the BM, and after apoptosis, cfDNA is released into the circulatory system, and some of it enters the urine through the filtration barrier of the glomeruli, thus also reflecting the disease status of the lesion location of MM patients [9, 10, 11]. The purpose of this study was to use 5hmC epigenetic marks in urine cfDNA to build a diagnostic model for early diagnosis of MM patients, build a differential diagnosis model for differential diagnosis of MM patients and AM patients, and based on the survival time at the last follow‐up of patients, conducted prognostic prediction for MM patients and searched for some biomarkers that predict the prognosis of MM.

The study results indicate that changes in 5hmC levels in gene functional regions are associated with MM, and 5hmC differential markers can be used for the diagnosis of MM. LINC00707 exhibits the most significant change in 5hmC levels in MM, and it can mediate a series of biological processes such as cell proliferation, apoptosis, metastasis, inflammation, and osteogenic differentiation [31]. Patients with MM are prone to develop peripheral neuropathy during treatment [32]. Genes involved in neurogenesis and axonal guidance are disrupted, and the frequency of axonal degeneration increases [33]. Proteoglycans, well‐known for their roles in cancer angiogenesis, cancer cell proliferation, and metastasis, are currently important targets for cancer immunotherapy [34, 35]. Calcium signaling pathway have been shown to be involved in the regulation of cell proliferation, migration and invasion, and are considered to be related to the prognosis of MM [36]. It is known that about 70%–80% of MM patients have myeloma bone disease, so the role of calcium signaling in the disease is self‐evident [37]. In addition, PI3K‐Akt, Wnt and MAPK signaling pathways have been reported to be involved in the proliferation and metastasis of MM cells, damage to osteoblast differentiation, induce drug resistance and immune escape of MM cells, and are closely related to the development of MM [38, 39, 40]. The importance of immunity and inflammation in the development of cancer is well known, and new approaches to treat diseases based on the human immune system are emerging [41]. The BM microenvironment of MM patients will be changed, and the levels of some antigens recognized by cellular immunity will be down‐regulated, which will lead to uncontrolled tumor proliferation and apoptosis resistance, and further promote tumor immune escape [42]. These results confirmed the reliability of the differential loci identified by 5hmC detection of urine cfDNA.

MM is a disease of the elderly, and its incidence is increasing with age, and statistical results show that the incidence of male is slightly higher than that of female [43]. Our results showed that older patients had higher scores, and men also had significantly higher scores than women, which was consistent with the existing research results. AM and MM are both malignant plasma cell diseases, that is, abnormal proliferation of clonal PCs exists in BM, and misdiagnosis is easy to occur in clinical diagnosis. Therefore, we constructed diagnostic and differential diagnostic models for MM based on 11 5hmC differential markers. Studies have shown that SFMBT2 can promote malignant proliferation of acute myeloid leukemia cells by regulating the miR‐582‐3p/ZBTB20 pathway [44]. ST3GAL1 gene can promote immune escape of tumor cells by targeting CD55 gene, and can promote tumor angiogenesis and disease progression [45, 46]. SCNN1B can inhibit tumor cell proliferation and colony formation by inhibiting c‐Raf, and further inhibit carcinogenic MEK–ERK signaling, thus playing a tumor suppressor role [47]. RPIA knockdown can induce cell senescence and increase the levels of p53 and p21 in cancer cells. Inhibition of RPIA expression can induce ROS levels and activate autophagy, apoptosis and cell senescence in cancer cells, which is a new idea for cancer treatment [48]. Together, these findings suggest that 5hmC markers derived from urinary cfDNA can serve as noninvasive diagnosis markers for MM patients.

The 5hmC differential markers based on urine cfDNA can also further predict the prognosis of MM. Genes that are upregulated in patients with low survival rates are enriched in more myeloid cell‐related pathways. Osteolytic lesions, bone pain, and fractures are one of the main clinical manifestations of MM, suggesting that the homeostasis of the BM environment is closely related to the prognosis of MM patients [49, 50]. Studies have shown that the FYN‐TRAF3IP2 fusion gene has an oncogenic effect in peripheral T‐cell lymphoma and induces the activation of the NF‐κB signaling pathway. FYN‐TRAF3IP2 has been proven to have a synergistic effect in the occurrence and development of lymphoma [51]. Moreover, the survival curve analysis of *TET2* showed that the survival time of patients decreased gradually with the down‐regulation of *TET2* expression, previous studies have shown that *TET2* gene mutations occur in MM patients, which may have an important impact on the survival time and treatment resistance of MM patients [52, 53]. At present, the existing clinical detection indicators are difficult to predict the prognosis of MM patients (Figure S6). In contrast, urine 5hmC markers have a higher predictive accuracy for the prognosis of MM patients and may provide new guidance for clinical medication use.

Nevertheless, this study still has some limitations. First, the sample sizes of controls and MM patients are small, and the diagnostic model, differential diagnosis model and prognostic predictive markers still need to be verified by larger sample cohorts. Second, China is not the country with the highest incidence of MM, but this study only enrolled Chinese patients and could not represent patients of other ethnic groups. Third, not all of the 5hmC differential markers obtained by the analysis have been reported to be associated with MM before, and their specific regulatory mechanisms remain to be experimentally investigated and verified. In the future, we will expand the sample size and dig deeper into the data to find more effective markers and build more stable and reliable models for MM diagnosis and prognosis prediction.

## 5 Conclusion

The 5hmC marker in urine cfDNA can be used for the diagnosis and prognosis prediction of MM. This is a non‐invasive and convenient method of liquid biopsy that could be very promising in the future.

## Author Contributions

**Weiwei Xie:** data curation (equal), writing – review and editing (equal). **Xuehui Li:** data curation (equal), methodology (equal), visualization (equal), writing – original draft (equal). **Hangyu Chen:** data curation (equal), methodology (equal), supervision (equal), writing – review and editing (equal). **Jinlin Chu:** data curation (equal), methodology (equal), validation (equal), writing – review and editing (equal). **Lei Zhang:** data curation (equal), formal analysis (equal), writing – review and editing (equal). **Bo Tang:** data curation (equal), writing – review and editing (equal). **Wenrong Huang:** data curation (equal), writing – review and editing (equal). **Linlin Li:** methodology (equal), writing – review and editing (equal). **Jian Lin:** conceptualization (equal), funding acquisition (equal), supervision (equal), writing – review and editing (equal). **Yujun Dong:** funding acquisition (equal), methodology (equal), writing – review and editing (equal).

## Ethics Statement

Written informed consent was obtained from all subjects in accordance with the Declaration of Helsinki and the study has been approved by the Ethics Committee of Peking University First Hospital (No. 2022 yan 196‐002).

## Conflicts of Interest

The authors declare no conflicts of interest.

## Supporting information


Figure S1.

Table S1.

Table S2.

## Data Availability

The data sets of this paper are included in the article and its additional files. The external dataset used and analyzed was obtained from the NCBI Gene Expression Omnibus (GEO) database, with the accession number GSE146649. (The download website is https://www.ncbi.nlm.nih.gov/geo/query/acc.cgi?acc=GSE146649). Ethics approval and consent to participate.
