# Supplementary material for: 5‐Hydroxymethylcytosine Profiles of cfDNA in Urine as Diagnostic, Differential Diagnosis and Prognostic Markers for Multiple Myeloma
Source: Cancer Med. 2024 Dec 23;13(24):e70477. doi: 10.1002/cam4.70477 (PMC11664239; doi:10.1002/cam4.70477)
Supplement: Supplementary file 1 — Figure S1. [file CAM4-13-e70477-s001.docx]

1. **Hydroxymethylcytosine Profiles of cfDNA in urine as diagnostic,** **differential diagnosis and** **prognostic markers for** **multiple myeloma**

Weiwei Xie^1^, Xuehui Li^3^, Hangyu Chen^2,4,5,6^, Jinlin Chu^3^, Lei Zhang^2,4,5,6^, Bo Tang^1^, Wenrong Huang^8^, Linlin Li^3,7^, Jian Lin^2,4,5,6^, Yujun Dong^1#^

1 Department of Hematology, Peking University First Hospital, No. 7 Xi Shi Ku Street, Xi Cheng District, Beijing, 100034, People's Republic of China.

2 Department of Pharmacy, Peking University Third Hospital, Beijing, China.

3 Department of Pharmacology, Xinjiang Medical University, Urumqi, China.

4 Synthetic and Functional Biomolecules Center, Peking University, Beijing, China.

5 Key Laboratory of Tropical Biological Resources of Ministry of Education, School of Pharmaceutical Sciences, Hainan University, Haikou, China.

6 Peking University Third Hospital Cancer Center, Beijing, China.

7 Key Laboratory of Active Components of Xinjiang Natural Medicine and Drug Release Technology, Urumqi, China.

8 Department of Hematology, Fifth Medical Center, General Hospital of The People's Liberation Army, Beijing, 100048, People's Republic of China.

Weiwei Xie, Xuehui Li, and Hangyu Chen are co-first authors.

#Corresponding authors:

Yujun Dong M.D.

Department of Hematology, Peking University First Hospital, No. 7 Xi Shi Ku Street, Xi Cheng District, Beijing, 100034, People's Republic of China. Phone: Tel +86-10-83575680. Email: [dongy@hsc.pku.edu.cn](mailto:dongy@hsc.pku.edu.cn)

Figure S1: The size and amount of urine cfDNA. (A) Healthy cohort urine sample quality as a result, cfDNA fragment size. (B) Multiple myeloma patients with urine sample
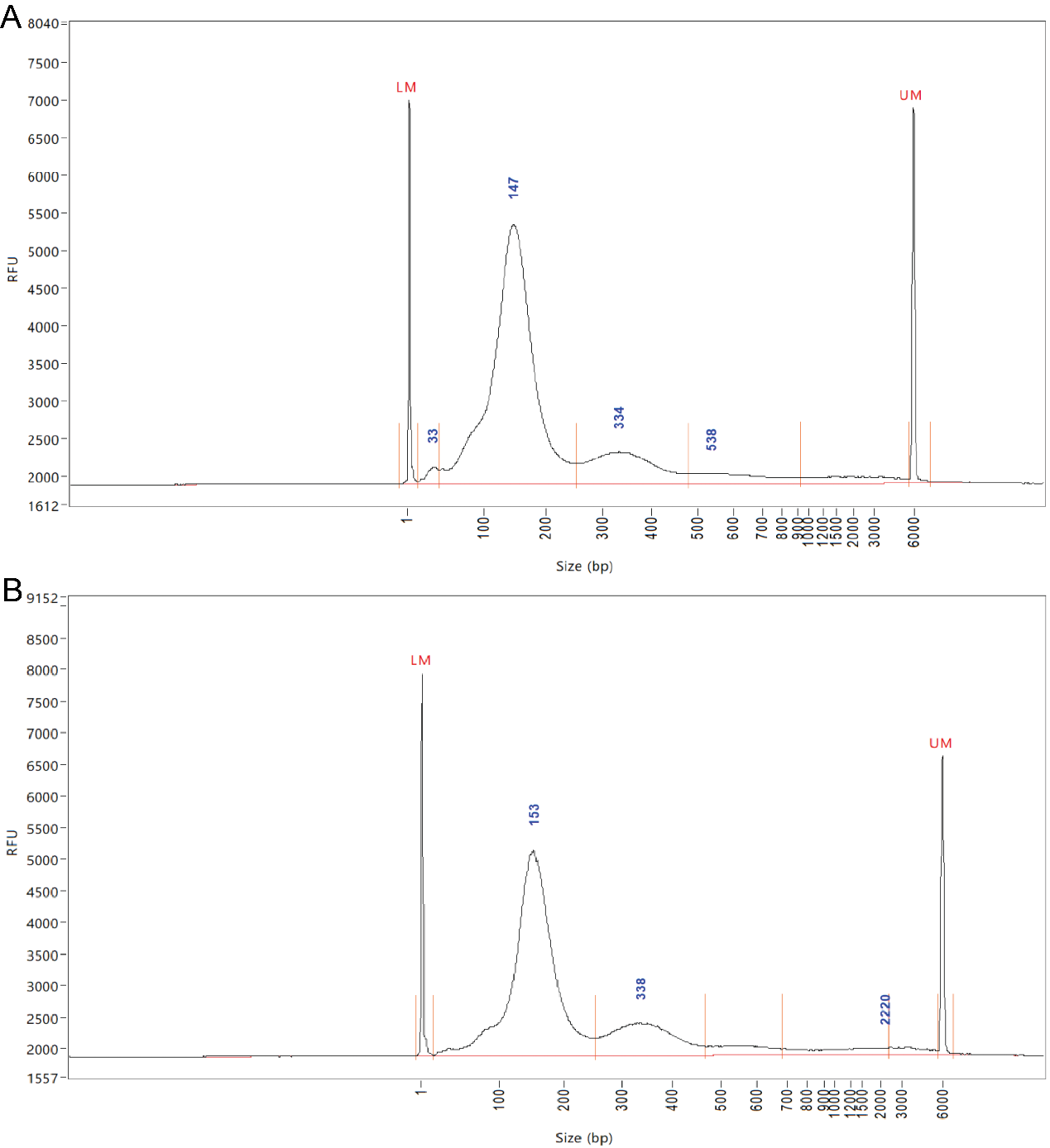
quality as a result, cfDNA fragment size.


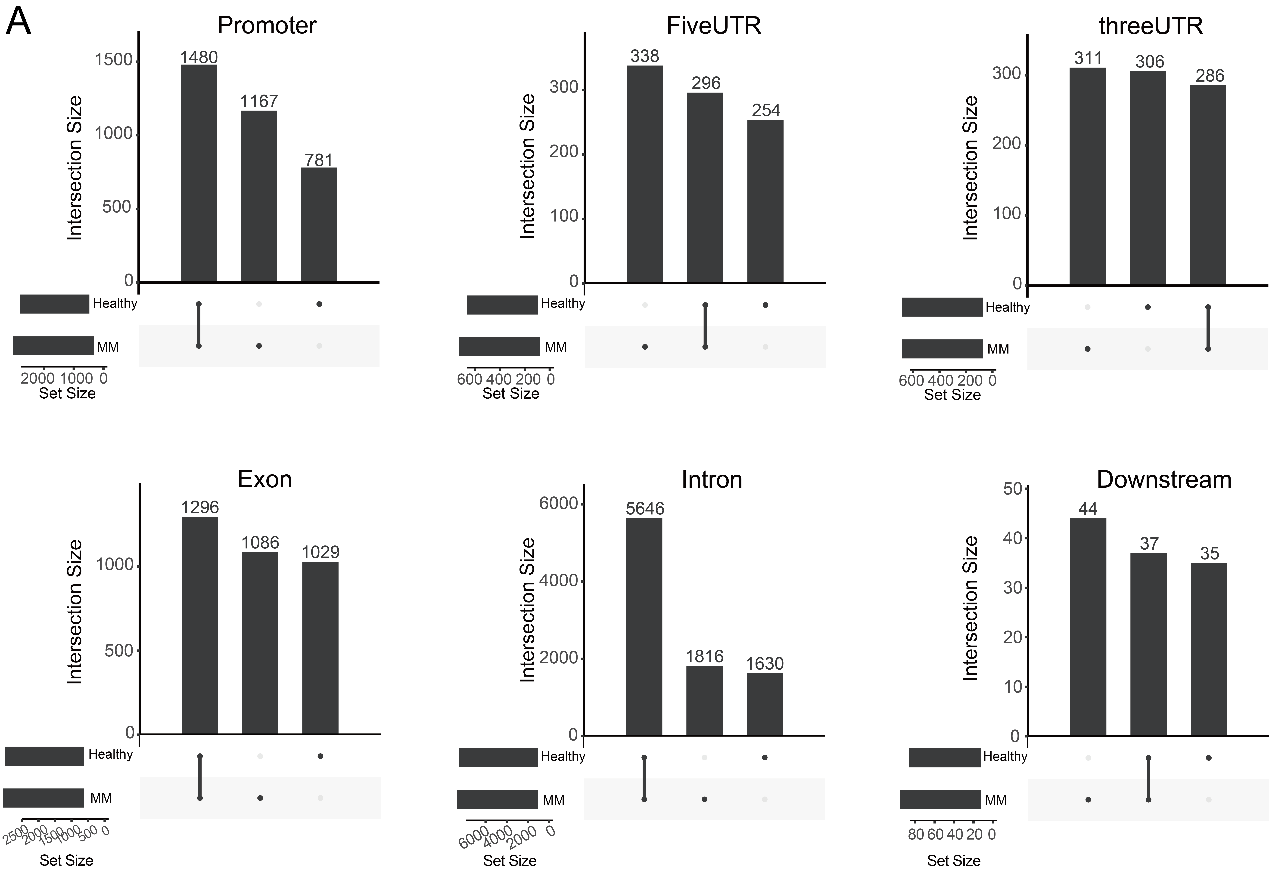
Figure S2: (A) Venn diagram. The 5hmC loci of healthy cohort and MM patients were mainly distributed in the region of the genome.


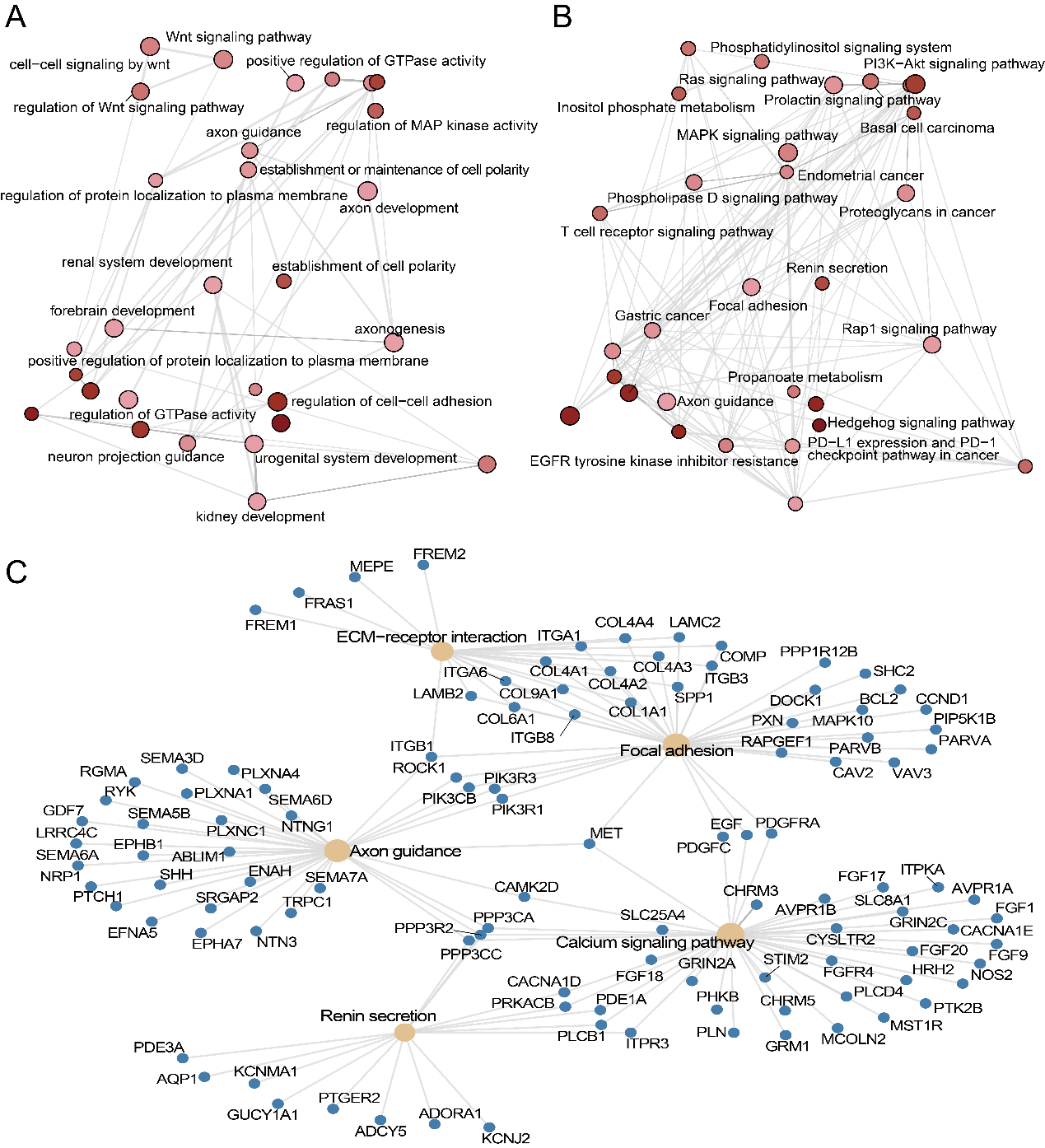
Figure S3: Functional annotation of differentially expressed genes between healthy cohort and MM patients. (A) Emapplot. GO enrichment analysis of all differentially expressed genes was performed. (B) Emapplot. KEGG enrichment analysis of all differentially expressed genes was performed. (C) CNET plot of all differentially expressed genes.


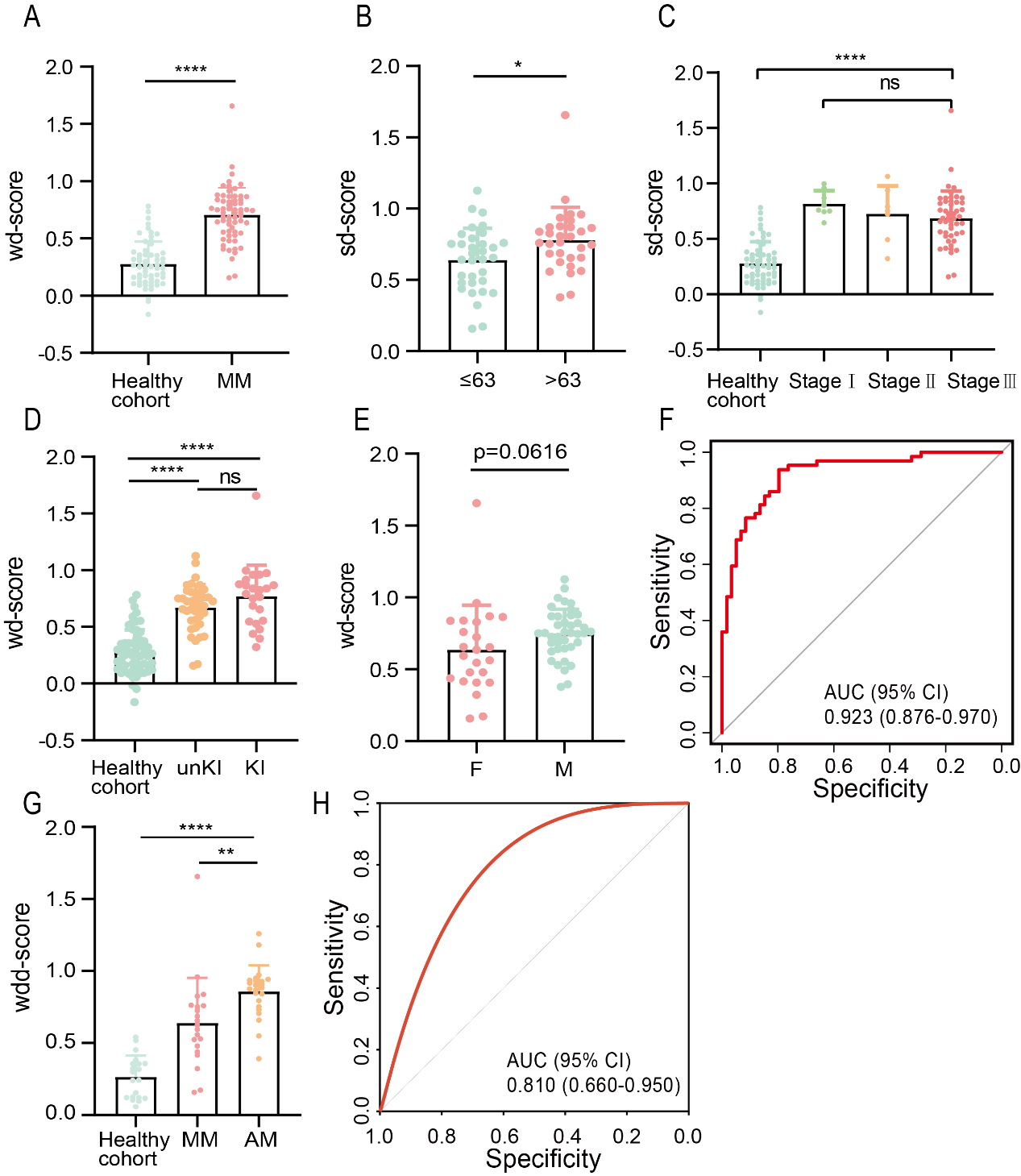


Figure S4: Box plot of weighted diagnostic scores (wd-score). (A) Comparison of wd-score between healthy cohort and MM patients. (B) Taking the median age of the sample (63 years) as the dividing line, the wd-scores of the two groups were compared. (C) Patients in stage I, II, and III were compared with the healthy cohort. (D) MM patients with and without renal injury were compared with healthy cohort. (E) Wd-scores were compared between men and women. (F) The weighted diagnostic score of markers was able to distinguish healthy cohort from MM patients with an accuracy of AUC=0.923. (G) The weighted differential diagnosis scores of healthy cohort, MM and AM in the differential diagnosis group were compared. (H) The weighted differential diagnosis score of markers could distinguish MM patients from AM patients, with an accuracy of 0.810 AUC.


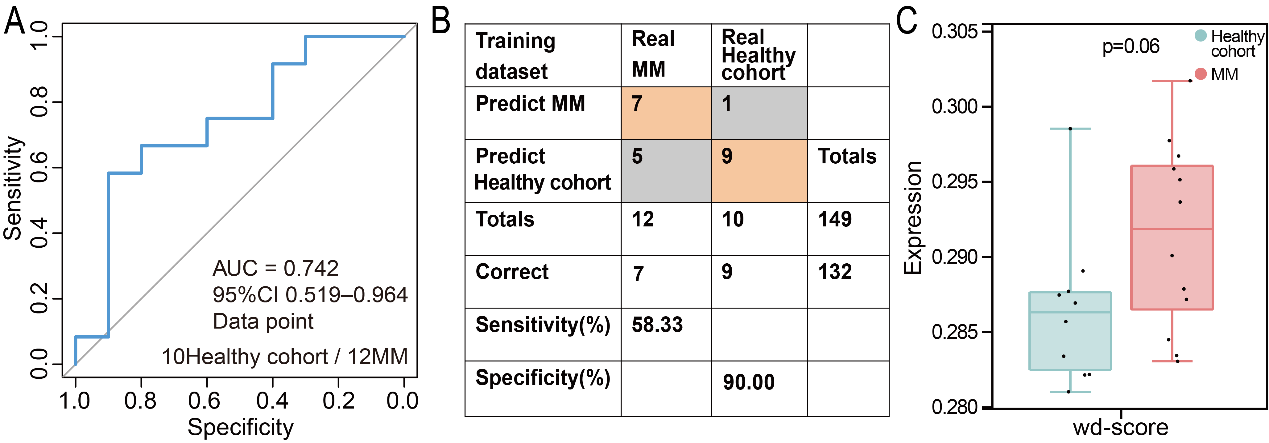


Figure S5: External dataset validation. (A) ROC analysis of the diagnostic accuracy of 11 markers obtained by machine learning in external dataset. (B) Specificity and sensitivity of the diagnosis of 11 markers in external dataset. (C) Comparison of wd-score between healthy cohort and MM patients..


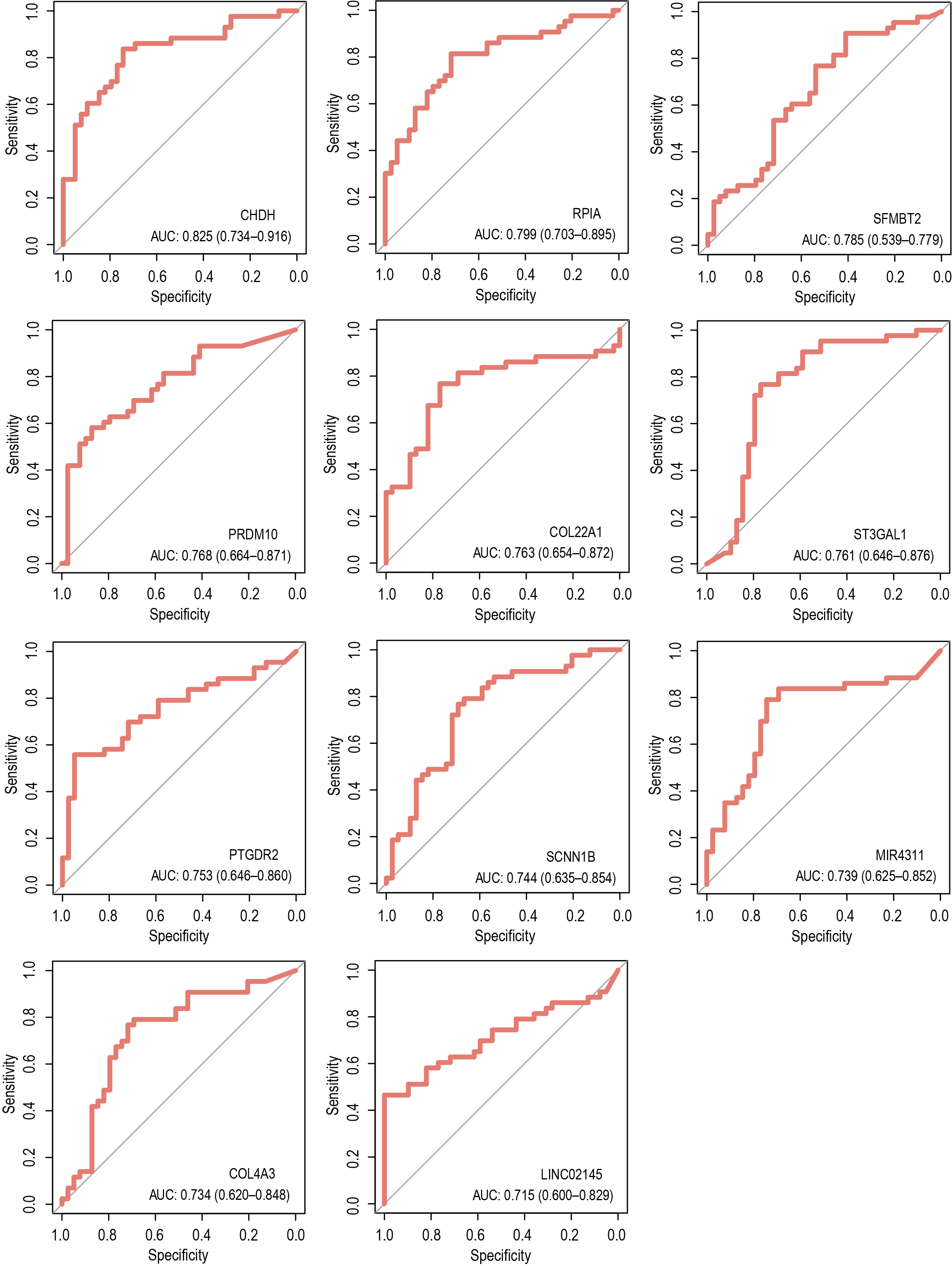


Figure S6: Separate ROC analysis of the 11 5hmC markers in the training group.


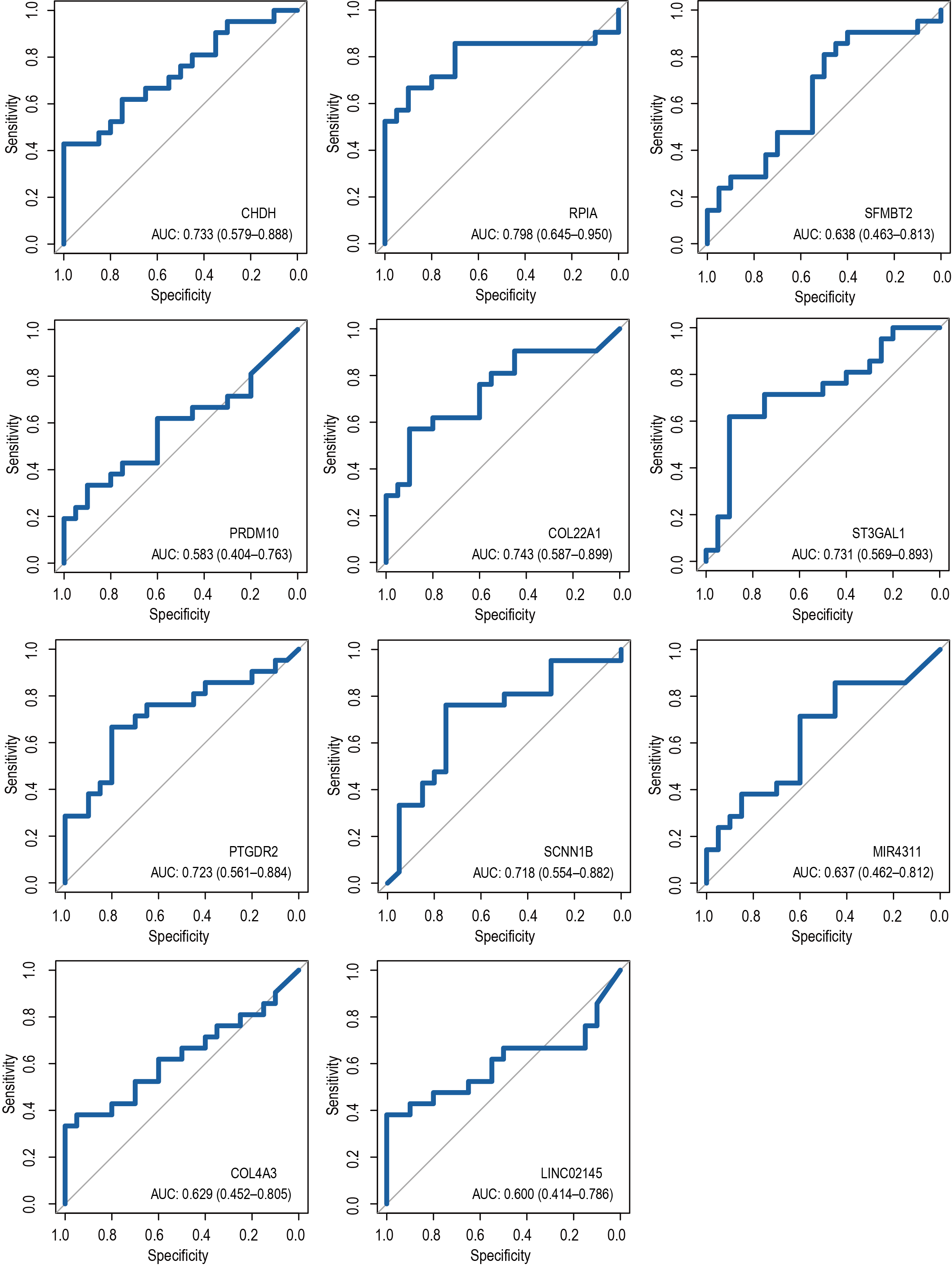


Figure S7: Separate ROC analysis of the 11 5hmC markers in the validation group.


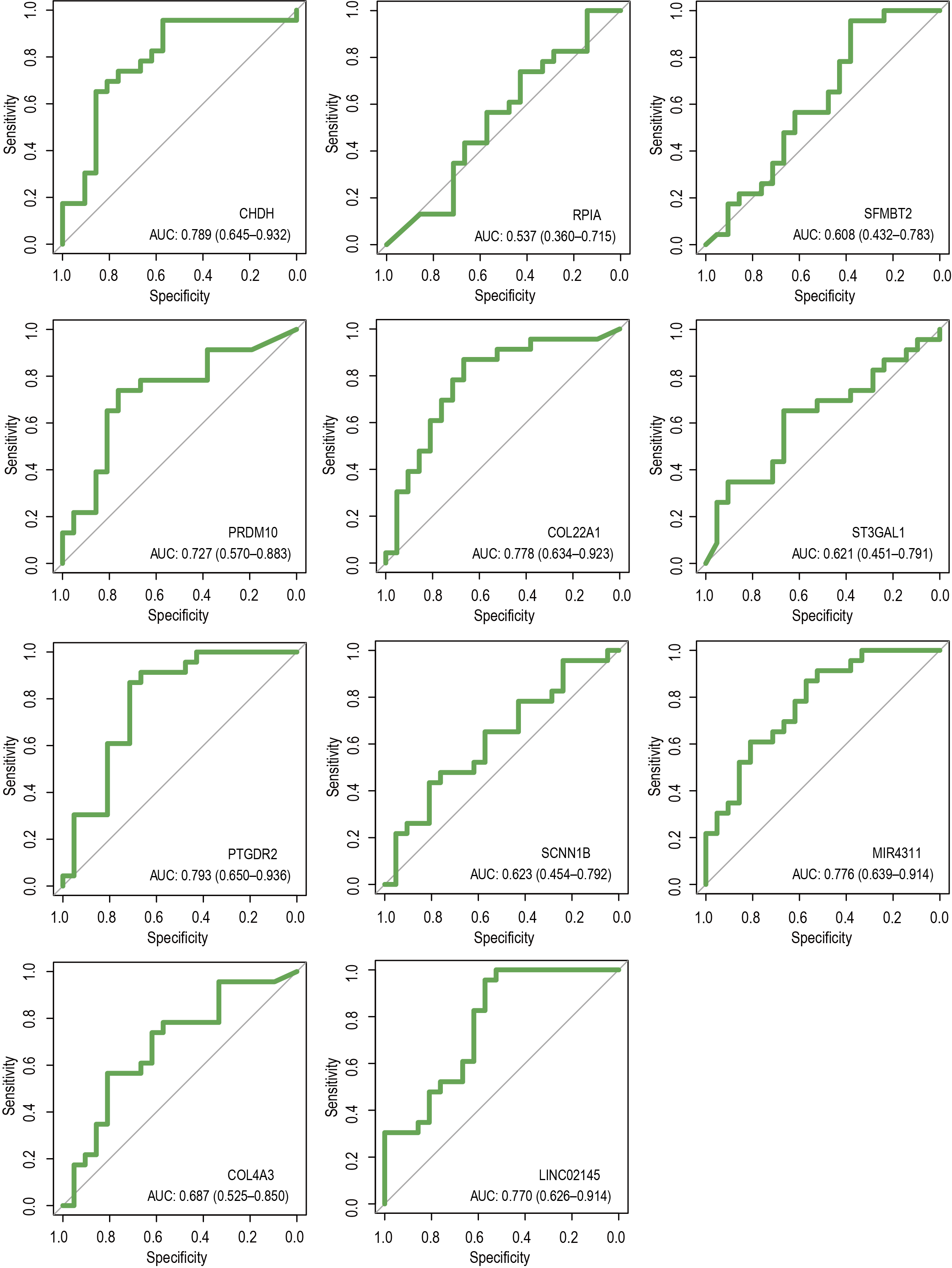


Figure S8: Separate ROC analysis of the 11 5hmC markers in the differential diagnosis group.


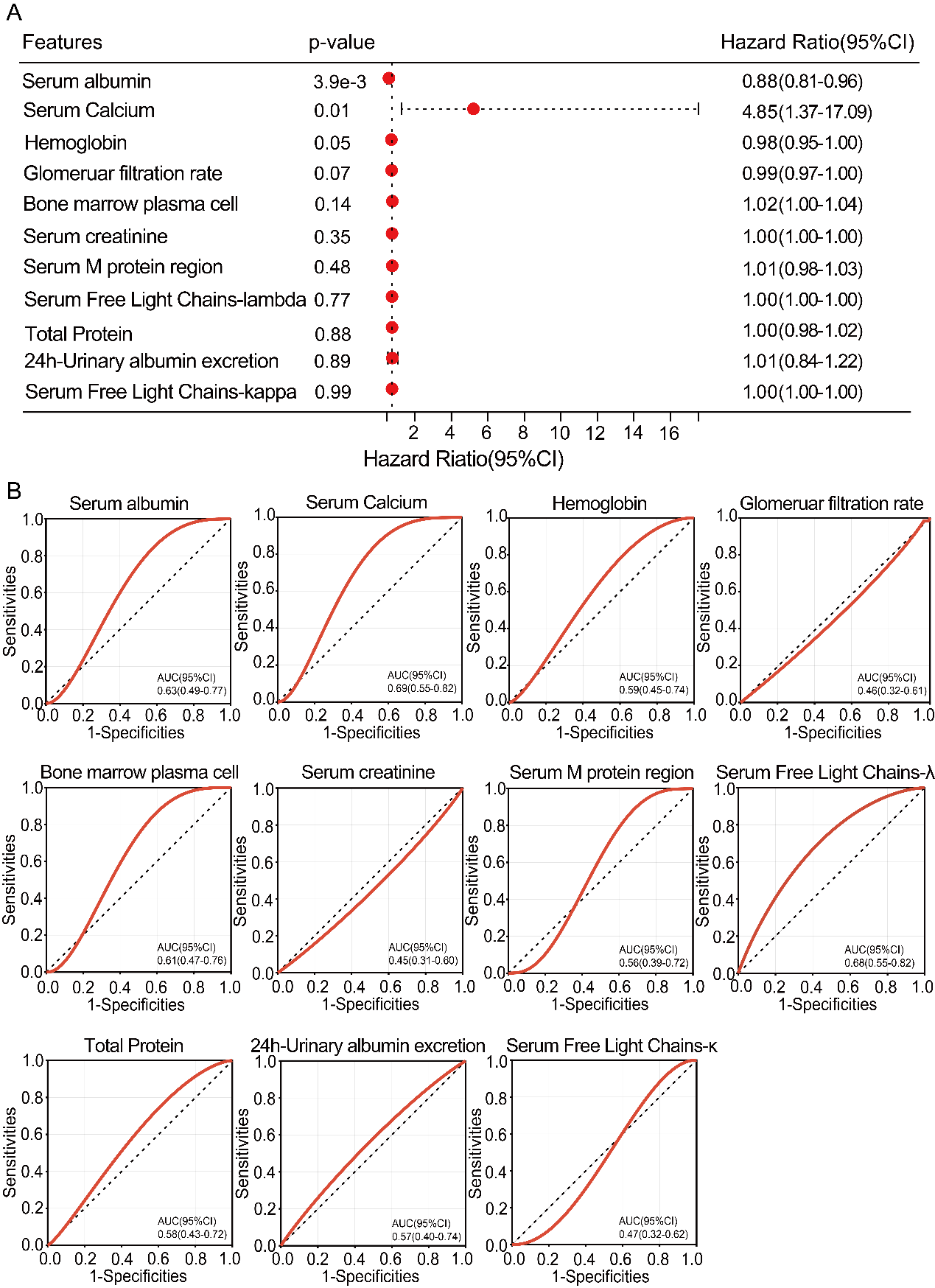
­­Figure S9: Prognostic prediction results of common clinical detection indicators Prognostic prediction results of common clinical detection indicators. (A) HR forest plot of common clinical detection indicators through multivariate Cox regression analysis. (B) Receiver operating characteristic (ROC) curve of commonly used clinical indicators for predicting prognosis.
